# A Rare Case of Atypical Hemolytic Uremic Syndrome (HUS) in an Adult Male: A Catastrophic Presentation

**DOI:** 10.7759/cureus.62590

**Published:** 2024-06-18

**Authors:** Sindhu C Pokhriyal, Samuel Sule-Saa, Jemima A Alemonai, Muthanna Mohammed Hasan Al-Ghuraibawi, Luckencia Pierre, Sunil Parkash, Kalpana Panigrahi

**Affiliations:** 1 Internal Medicine, One Brooklyn Health-Interfaith Medical Center, Brooklyn, USA

**Keywords:** complement, hus, streptococcus pneumoniae, atypical, syndrome, uremic, hemolytic

## Abstract

Atypical hemolytic uremic syndrome (HUS) is extremely rare in adults. HUS is characterized by hallmark features of microangiopathic hemolytic anemia, thrombocytopenia, and acute renal injury. Atypical HUS (aHUS) is caused by uncontrolled complement activation. The complement activation can be triggered by infections such as *Streptococcus pneumoniae* or influenza, pregnancy, malignancy, cytotoxic drugs, organ transplants, or autoimmune diseases. Genetic mutations and autoantibodies have been found to play a crucial role in the pathogenesis of dysregulated complement activity. The majority of cases of atypical HUS due to invasive *S. pneumoniae* infection are more commonly seen in children. We present a case of *S. pneumoniae* HUS (Sp-HUS) presenting with multiorgan failure, disseminated intravascular coagulation (DIC), and limb ischemia in an adult. This case highlights the importance of considering *S. pneumoniae* HUS (Sp-HUS) in the differential diagnosis of thrombotic microangiopathies (TMA) in adults.

## Introduction

Thrombocytopenia, acute renal failure, and intravascular hemolysis are the hallmarks of hemolytic uremic syndrome (HUS), one of the thrombotic microangiopathies (TMA) [1]. Hemolytic uremic syndrome (HUS) can be classified into three categories: typical HUS, which is caused by an infection from Shiga toxin-producing *Escherichia coli* (STEC); atypical HUS (aHUS), which is typically caused by genetic defects in the complement system; and secondary HUS, which occurs alongside another underlying condition [2]. The principal factors that are associated with atypical HUS (aHUS) include *Streptococcus pneumoniae* infections, influenza virus [3], transplantation [4-7], autoimmune disease, cancer, pregnancy, and the administration of specific cytotoxic medications [8-13]. The organ damage resulting from STEC-HUS is attributed to the cytotoxic impact of Shiga toxin, while the organ damage associated with aHUS is typically produced by complement-mediated mechanisms [14]. With an annual incidence of 0.06 cases per 100000 children in the USA, invasive pneumococcal infection is the primary cause of aHUS that has been reported in the pediatric population. Only 10 instances of *S. pneumoniae HUS* (Sp-HUS) have been reported in the adult population so far [15-18].

Although it tends to occur in children, adult cases are rare and might offer diagnostic difficulties because of clinical symptoms that are shared by other thrombotic microangiopathies. We present a case of aHUS in an adult male to emphasize the importance of the timely recognition and management of this condition.

## Case presentation

A 47-year-old male with a past medical history of polysubstance use disorder presented to the emergency department with a one-day history of diarrhea, shortness of breath, altered mental status, and anuria. On examination, he was febrile, dehydrated, tachypneic, tachycardic, agitated, and drowsy. Laboratory investigations revealed thrombocytopenia (platelet of 112000/µL), intravascular hemolysis (hemoglobin {Hb} of 12 g/dL and schistocytes in peripheral smear), reticulocyte count of 6.8%, renal failure (blood urea nitrogen {BUN} of 23 mg/dL and creatinine of 3.4 mg/dL), and elevated C-reactive protein (CRP) (15 mg/dL), procalcitonin (273 ng/mL), lactate dehydrogenase (LDH) (733 IU/L), haptoglobin (less than 10 mg/dL), creatine kinase (CK) (693 U/L), and troponin (1565 ng/L).

Within a few hours of admission, the patient's condition deteriorated. There was an abrupt worsening of intravascular hemolysis (Hb of 11.1 g/dL and platelet of 30000/µL) and renal function (BUN of 35 mg/dL and creatinine of 5.5 mg/dL); an increase in bilirubin (total bilirubin of 4.8 mg/dL and direct bilirubin of 3.1 mg/dL); a drastic rise in liver enzymes, both alanine aminotransferase (ALT) and aspartate aminotransferase (AST) of >1000 U/L; and an abnormality of the coagulation-fibrinolytic system (prothrombin time/international normalized ratio {PT/INR} of 30/2.6, partial thromboplastin time {PTT} of 75, and D-dimer of >69000 ng/mL fibrinogen equivalent units). The PLASMIC score for TTP was less than 4; thus, thrombotic thrombocytopenic purpura was unlikely.

**Figure 1:**
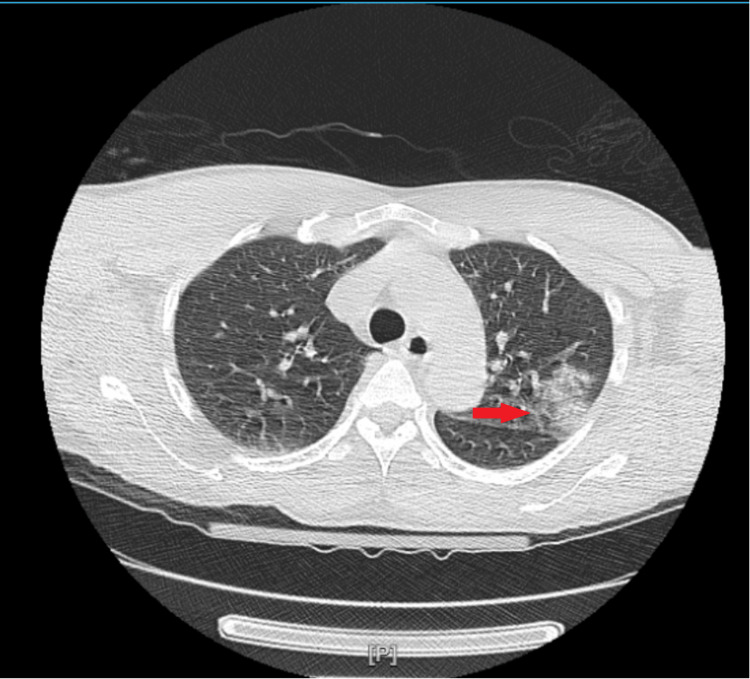
CT scan of the chest showing the red arrow pointing at the left upper lobe consolidation

The patient was treated with broad-spectrum antibiotics, continuous renal replacement therapy (CRRT), and other supportive treatments after a provisional diagnosis of sepsis with multiorgan failure was made. Left upper lobe consolidation was seen on a CT scan of the chest (Figure 1), and *Streptococcus pneumoniae* was grown in the blood culture. Further investigations showed reduced C3 (51 ng/dL) and normal C4 (16 mg/dL). The venous and arterial duplex study of the lower extremities demonstrated both arterial and venous occlusion, and the patient unfortunately developed gangrene of the extremities. Complement factor H antibody titers sent were positive. The provisional diagnosis was revised to hemolytic uremic syndrome secondary to invasive pneumococcal disease (IPD). He subsequently developed mummification of his hands, feet, nose, and ears. Unfortunately, the patient's clinical status worsened, and the family opted for palliative extubation. He ultimately succumbed to the illness.

## Discussion

Atypical hemolytic uremic syndrome is an extremely rare but potentially fatal disorder characterized by the dysregulation of the alternative complement pathway. Although complement dysregulation and genetic alterations are frequently linked, the precise etiology is yet unknown [2]. Some studies however suggest that it is triggered by infections (usually respiratory tract infections) and is associated with a genetic or acquired defect in the regulation of complement activation on host cells [3-13]. In order to avoid end-organ damage and achieve better results, prompt diagnosis and treatment are essential. By focusing on complement-mediated hemolysis and thrombotic microangiopathy, eculizumab has fundamentally changed the way that aHUS is managed [18].

Knowing that *Streptococcus pneumoniae* is the most prevalent cause of adult lobar pneumonia and that there may be a connection between drug abuse and invasive pneumococcal disease (IPD) is crucial for clinicians. According to studies, those who use drugs including alcohol, cigarettes, and opioids are more likely to get invasive pneumococcal disease (IPD). These drugs have the potential to compromise the body's defenses against infections and weaken the immune system, which increases a person's susceptibility to pneumococcal infections [19].

Our patient presented with fever, anuria, altered mental status, multiorgan failure, features of disseminated intravascular coagulation (DIC), peripheral microvascular thrombosis, and venous thrombosis. Laboratory findings indicate the presence of thrombotic microangiopathy (TMA) including low platelets, hemolytic anemia, and renal failure. The low C3 levels and normal C4 levels along with positive complement H antibody titers strongly indicate the activation of the alternative pathway, thus suggesting a diagnosis of atypical hemolytic uremic syndrome (aHUS). About 10%-20% of patients with aHUS may present with the extrarenal manifestation of aHUS, which can include altered mental status just like our patient [18].

Clinical deterioration may be associated with the transfusion of unwashed packed cells and platelets. The transfusion of unwashed packed cells can cause transfusion-associated hemolysis, and platelets contribute to the formation of blood clots, which is the main pathology in aHUS.

The recommended therapies to manage atypical hemolytic uremic syndrome (aHUS) include plasma exchange or plasma infusion to replace the deficient complement regulatory factors. These therapies are effective in improving thrombotic microangiopathy (TMA) and reducing the risk of additional complications. Another recommended treatment is eculizumab, which works by suppressing the cleavage of C5 to C5a and C5b, thereby preventing the production of the membrane attack complement complex (MAC). This can help to alleviate the symptoms and prevent further damage associated with aHUS.

Initiating fresh frozen plasma (FFP) and eculizumab and avoiding unwashed pack cell and platelet transfusion may alter the patient's clinical outcome.

## Conclusions

This case highlights the importance of considering atypical hemolytic uremic syndrome in the differential diagnosis of thrombotic microangiopathies in adults. Prompt diagnosis and eculizumab-based targeted therapy can improve prognoses and prevent disease progression. Further research is needed to understand the underlying pathogenesis of aHUS and to help with early diagnosis in order to tailor treatment plans for adult patients.
